# The development of Anthropocene Awareness Scale

**DOI:** 10.1371/journal.pone.0316315

**Published:** 2025-02-06

**Authors:** Donghun Kang, Moon Choi

**Affiliations:** 1 Graduate School of Science and Technology Policy, Korea Advanced Institute of Science and Technology, Daejeon, South Korea; 2 Graduate School of Data Science, Korea Advanced Institute of Science and Technology, Daejeon, South Korea; Uninettuno International Telematic University, ITALY

## Abstract

The political influence of the Anthropocene concept stems from its analytic potential to encompass various disciplines and capture public attention. However, there is a lack of research examining the extent to which the public has adopted the views embodied in the Anthropocene. We developed a scale to measure awareness of the Anthropocene. Based on a thorough review of Anthropocene studies, an initial set of fifteen items was generated to develop the scale. These items were then subjected to an empirical test, using a sample in South Korea (N = 1,668; aged 19 to 90). After a series of reliability and factor analyses, the Anthropocene Awareness Scale was optimized into a unidimensional scale comprising eight items (Cronbach’s alpha = 0.88). Designed to explore individual attitudes, the scale could provide quantitative researchers with an entry point into the Anthropocene discourse and facilitate empirical studies that generate evidence for environmental policies and education in the Anthropocene.

## 1. Introduction

The concept of the Anthropocene was first proposed by Earth system scientists and geologists led by Nobel prize-winning atmospheric chemist Paul Crutzen [1]. These scientists argued that human activity has become the fundamental cause of global environmental change, suggesting a new geological epoch called the Anthropocene. The discussion of the Anthropocene has since rapidly spread to humanities and social sciences fields, including history, philosophy, art, politics, sociology, geography, and anthropology [2–5].

The concept of the Anthropocene differs from other existing environmental and ecological discussions [6]. First, the Anthropocene gives rise to a different way of thinking about humans and the Earth. The Anthropocene is the epoch in which humans have reshaped the Earth; thus, humans are “planet shapers” in the Anthropocene [7]. Yet, the Anthropocene encompasses more than the increasing human impacts on ecosystems. The Anthropocene is a term that applies to the Earth system. The concept of the Earth system emerged in the 1980s and 1990s, with the development of Earth system science [8]. Ecological thinking, based on the biological science of how organisms interact with their local environments, emerged in the 1960s and 1970s. Ecology studies the local, but Earth system science studies the Earth as a whole system [9]. This concept was designed to capture the qualitative leap from disturbances of ecosystems to disruption of the Earth system [6]. Second, the crisis in the Anthropocene is planetary-scale, irreversible, and nonlinear, foretelling the catastrophic extinction of humans on Earth [10]. The discourse surrounding the Anthropocene, the awareness of the scope and urgency of this crisis, aligns with new discussions and practices of contemporary environmental politics emerging from recognizing a global ecological crisis that threatens humanity’s existence [11].

Given the present complexity and future uncertainty, it is impossible to find one clear solution in the Anthropocene [12]. Davis and Turpin [13] emphasize that cooperation between natural and social sciences and collaboration with researchers and diverse groups, such as journalists, artists, activists, and citizens, are actively encouraged. The Anthropocene concept is adaptable and has resonated across disciplines and the public. Previous research has advocated for its active utilization [14, 15]. As stated by Hecht [16], “Nomenclature does political work; a single word can create a discursive infrastructure for political change.” (p. 116). Hecht [16] also emphasized that words have power only when they are widely accepted, and the political influence of the Anthropocene concept resides in its analytical potential to bridge disciplines and foster collaboration across fields.

While the Anthropocene concept has experienced broad diffusion across academic fields and public discourse, little research has investigated the public’s adoption of its central tenets. A survey instrument designed to explore individual attitudes could provide quantitative researchers with an entry point into the Anthropocene discourse and facilitate empirical studies that generate evidence for environmental policy and education in the Anthropocene. Existing measures assessing environmental attitudes have limitations in capturing the theoretical backgrounds, terminology, and critical urgency associated with the Anthropocene. Developing a new scale grounded in the Anthropocene could unlock the concept’s adaptability and analytical potential. Therefore, we conducted a thorough review of core literature in Anthropocene studies to gather ideas for potential items and develop a scale to measure awareness of the Anthropocene. Such a scale will allow future researchers to examine empirically the degree to which individuals accept facets of the Anthropocene.

## 2. Literature review

### 2.1. Determining the construct

Before developing the content of a scale, researchers need to clarify the construct being measured. The purpose of scales is to measure a construct that is difficult to define and cannot be obviously observed [17]. There are many scientific steps in developing a reliable and valid scale, but there is no clear criterion for comparing the performance of scales that measure such elusive phenomena [18]. Thus, it is essential that the scale reflects theories related to the construct. The clarity of the scale is based on theories, and consideration of relevant theories must take precedence for developing a scale [17].

The Anthropocene Awareness Scale measures the extent of awareness regarding the Anthropocene. Previous research has developed scales related to environmental attitudes [19–22]; however, these scales are different from the construct of the Anthropocene awareness, in particular in terms of the level of specificity. Existing scales such as the Ecological Attitudes and Knowledge Scale [19], Environmental Concern Scale [20], New Environmental Paradigm Scale [21], and New Ecological Paradigm (NEP) Scale [22] were designed to measure the attitudes and knowledge based on environmental discussion of their times; in contrast, the Anthropocene Awareness Scale that we propose in this study is to measure more specific constructs related to the Anthropocene. To articulate the purpose of the scale clearly, we conducted a literature review on the Anthropocene, including a discussion on the causes, current situation, and proposed alternatives to the Anthropocene.

### 2.2. Facets of the Anthropocene

First of all, the increasing impact of humans is comparable to nature, and humans have geological force in the Anthropocene. Humans have a dominant impact not only on the local ecosystem but also on the Earth system. The underlying assumption of the Anthropocene is that humans, as planet shapers, have reshaped the Earth [7]. The proposition that humankind is the main cause of the predicaments of the Anthropocene, such as climate change, contradicts the modern nature-society dichotomy that human society and the natural environment exist separately [3].

Over the past 70 years, human socioeconomic activities, such as population growth, urbanization, energy, and fertilizer use, have increased rapidly in the world, and the Earth system has drastically changed at the same time in terms of carbon emission, marine acidification, and destruction of the ozone layer. Steffen et al. [5] argued that the rapid increase in human activity has directly led to the current planetary crisis and called the phenomenon the Great Acceleration. The concept of the Great Acceleration received widespread support, and most of the Anthropocene Working Group voted to mark the mid-twentieth century as the beginning of the new epoch [23].

The Anthropocene represents an unprecedented planetary crisis. Rockström et al. [4] identified nine elements of the Earth system, including climate change, biodiversity, and the stratospheric ozone layer, and suggested thresholds for each, planetary boundaries that must be maintained to ensure the stability of the Earth system. Some of the nine elements have already gone outside the safe boundaries for humanity, and others are close to exceeding the limits [5]. Once elements exceed these thresholds, the planetary crisis is nonlinear, irreversible, and unpredictable. The crisis in the Anthropocene is entirely different from the crisis in the Holocene [24]. While the ecological crisis was regarded as a resilient, circular crisis that risk management strategies could control, the planetary crisis is an irreversible crisis that cannot be predicted and controlled at the tipping points.

The discussion of the Anthropocene as a rupture implies an unprecedented catastrophe. The crisis of the Anthropocene, characterized by the planetary-scale and planetary boundaries or tipping points, is full of uncertainty, so we can neither investigate a cause nor find a solution [15]. The Earth system changes regardless of human predictions or intentions and threatens society and civilization. The predicaments of the Anthropocene, such as air pollution making it hard to breathe and heat waves breaking the record every year, cause anxiety about the future through intense physical experience. Compellingly, this situation suggests that a sixth extinction of species, this time including humans, may occur [10].

On the other hand, discourse on the Anthropocene finds the catastrophe caused by human activities rooted in the notion of modernity. The structures and systems in a modern society designed for the pursuit of liberty and happiness drive into catastrophe, threatening the survival of all human beings [24]. The Anthropocene is connected to reflections on modernity discussed in the humanities and social sciences [6, 25]. The discussion of the Anthropocene considers scientific technology, economic growth, and anthropocentrism, the basis of modernity, as the fundamental causes of the catastrophe. Humans have had a dominant impact on nature through science and technology in modern history while destroying other living things and disturbing the Earth system at unprecedented speed and scale [26].

Scholars based on new materialism believe that anthropocentrism based on nature-society dualism is the fundamental cause of the crisis in the Anthropocene [15, 27]. Anthropocentrism regards nature as a passive object waiting for human intervention and humans as rulers who have the right to control nature for their purpose. Anthropocene scholars argue that humans must abandon modern dualism, which separates humans from nature and grants them special privileges. They espouse the modest attitude that humankind is a species with its own capabilities and limitations rather than a ruler of nature.

Solutions for the Anthropocene have been the subject of heated debate. Ecomodernists emphasize that human social, economic, and technological capabilities can improve all human lives, stabilize climate change, and protect the natural world [28]. They say we must embrace the possibility of modifying the Anthropocene through human capacity. They believe that the planet’s crisis might be a new opportunity to show humans’ ability to alter and control the Earth system. One example is geo-engineering, which assumes that climate change can be solved by manipulating the climate through science and technology.

However, other scholars of the Anthropocene argue that we should break with modernity represented by science and technology, economic growth, and anthropocentrism, think of the Anthropocene as a rupture, and seek a new planetary future [9]. Given the self-destructive nature of modernity and the uncertainty of the planetary crisis, existing norms, practices, and systems are not enough to overcome the Anthropocene, and unconventional and unexpected solutions are needed [3, 15, 27, 29]. As Latour [30] posited, "the name of this new geohistorical period may become the most pertinent philosophical, religious, anthropological, and—as we shall soon see—political concept for beginning to turn away for good from the notions of modern and modernity" (p. 116).

While existing environmental studies focus on coming up with solutions through markets and technology, the Anthropocene studies focus on various explorations to understand the present and to imagine the future [3]. Given the present complexity and future uncertainty, seeking a single clear solution becomes neither easy nor meaningful. Instead, epistemological experiments are necessary to explore various connections and trajectories. Scholars based on new materialism intend to dismantle nature-society dualism—the fundamental cause of the Anthropocene—and transform the relationship for cooperation among various species, including humankind. They emphasize the relational ontology. Humans and nonhumans, including animals, plants, things, and machines, make up the world together—an assemblage. This creates a multi-species kinship, an attunement among humans and nonhumans [31, 32]. For example, Tsing [25] demonstrates that various lives are intertwined through the production and consumption of mushrooms, Lorimer and Driessen [33] propose a rewilding and wild experiment, a new conservation method focusing on more-than-human, and Van Dooren [34] seeks new ethical practices, an entanglement of human and nature through various birds.

## 3. Research design and methods

### 3.1. Scale development

#### 3.1.1. Generating an item poo

After defining the construct of interest and the purpose of the scale, the next step is to develop an initial item pool relevant to the construct. The desired result is that the content of each item will reflect the latent variable, and all items will constitute a homogeneous scale measuring the construct of the interest. Based on the literature review in the previous section, we generated items reflecting facets of the Anthropocene, such as the Earth system, the Great Acceleration, planetary boundaries, the sixth extinction, and multispecies relations. We focused on capturing the theoretical backgrounds, terminology, and critical urgency associated with the Anthropocene to supplement existing measures.

First, we considered the underlying assumption of the Anthropocene. The Anthropocene gives rise to a different way of thinking about humans and the Earth. Humans have reshaped the Earth, and humans are “planet shapers” in the Anthropocene [7]. The concept was designed to capture the qualitative leap from disturbances of ecosystems to disruption of the Earth system [6]. Humans have a dominant impact not only on the local ecosystem but also on the Earth system. The rapid increase in human activity has directly led to the current planetary crisis called the phenomenon of the Great Acceleration [5].

Second, we reflected on the features of the urgent crisis in the Anthropocene. The predicament in the Anthropocene is the unprecedented planetary crisis. The planetary crisis is nonlinear, irreversible, and unpredictable if critical thresholds are exceeded [4]. While the ecological crisis was regarded as a resilient, circular crisis that risk management strategies could control, the planetary crisis is irreversible and cannot be predicted and controlled at the tipping points. The Earth is on the brink of sixth extinction, a massive loss of living species, including humans [10].

Third, we included a discussion of the causes of and proposed alternatives to the Anthropocene. The discussion of the Anthropocene considers scientific technology, economic growth, and anthropocentrism, the basis of modernity, as the fundamental causes of the catastrophe [26]. Scholars based on new materialism believe that anthropocentrism based on nature-society dualism is the fundamental cause of the crisis in the Anthropocene [15, 27]. They intend to dismantle nature-society dualism and transform the relationship for cooperation among various species, including humankind. They emphasize a modest attitude that humankind is a species with its own capabilities and limitations rather than a ruler of nature.

We generated 15 items in consideration of relevant redundancies. An initial item pool should be more expansive because models of scale development are based on redundancy [17], in order to attain good internal consistency reliability. Having many items is a preventative measure for poor internal consistency. A larger item pool is generally preferable, but determining the precise number of items to include in an initial pool can be challenging. First, it is important to avoid irrelevant redundancies. Reliability will be inflated if items are redundant with their incidental vocabulary and grammatical structure. Cronbach’s alpha cannot distinguish between covariance due to irrelevant redundancies and covariance due to the influence of the latent variable. Second, it may be difficult to generate a large pool of items appropriate to measure the construct of interest in specific content areas. Survey overload, characterized by excessive items, can induce fatigue and concentration issues in respondents, compromising their responses and potentially biasing results [17]. Empirical data may suggest that a relatively small number of items are sufficient to achieve good internal consistency. Some previous studies have employed an initial item pool size only 50% larger than the final scale [35–38].

To reduce complexity and increase clarity, we eliminated lengthy items without sacrificing the meaning of an item. The number of words and syllables per sentence affects reading difficulty level. The target scale aims to reach a reading level of sixth grade for use with the general population. A sixth-grade-level sentence typically has 16 words and 20 syllables [17]. In addition, we avoid multiple negatives ambiguous pronoun references. S1 Table shows the initial item pool with 15 items for the Anthropocene Awareness Scale.

#### 3.1.2. Determine the format for measurement

Scale developers should decide on the appropriate forms of scale items, such as the Likert scale, semantic differential, visual analog, and numerical response formats. Including many scale items increases variability, and a response format with more options than a binary response format might provide more helpful information. At the same time, the respondents should be able to distinguish response options meaningfully. The specific wording and physical placement of response options can help the respondents to distinguish meaningfully between them.

In this study, a 5-Likert scale is used for measuring the perception of the Anthropocene. Likert response formats are commonly used in questionnaires that measure opinions, beliefs, and attitudes [17]. A Likert scale item typically presents a declarative statement, followed by a series of response options indicating the respondent’s agreement level. The response options should be worded so that the intervals between them represent roughly equal levels of agreement.

#### 3.1.3. Administering items to a development sample

The sample size for scale development should be large enough to produce reliable scale estimates. Additionally, to ensure that the scale can be applied to the population of interest, the sample should be representative of the population. The patterns of covariation among the items are not stable with a small sample size. Nunnally [39] suggests 300 subjects are an appropriate number, and DeVellis [17] shows that fewer than 300 people might be sufficient for a single scale with 20 items. The data analyzed in this study was primary data from an online survey carried out in South Korea in October 2021. The participants were recruited via a quota sampling by region, gender, and age group. The sample consisted of 1,668 persons aged 19 to 90 recruited from all sides of the country with 50.4% of women. The KAIST Institutional Review Board (IRB) approved this study.

#### 3.1.4. Evaluating items and optimizing scale length

The optimal trade-off between reliability and brevity is one of the critical issues in scale development. Scales with more items are generally more reliable than scales with fewer items. However, scales with fewer items are generally less burdensome for respondents to complete than scales with more items, which may lead to higher response rates and more accurate data [17].

Correlation, reliability, and factor analysis are useful for evaluating the performance of the individual items that constitute the scale. First, the corrected item-total correlation shows how intercorrelated the items are. An item with a high correlation value is more desirable than one with a low correlation value, as it indicates that the item is more strongly related to the overall scale and is, therefore, more likely to be a reliable measure of the construct. Second, Cronbach’s alpha, or coefficient alpha, is a widely used measure of internal consistency reliability and one of the most important indicators for evaluating items. Alpha measures the proportion of variance in the scale scores attributable to the underlying construct or true score. DeVellis [17] suggests the following ranges for research scales: between 0.65 and 0.70, minimally acceptable; between 0.70 and 0.80, respectable; between 0.80 and 0.90, excellent; and much above 0.90, where one should consider shortening the scale. Third, factor analysis shows whether the scale is unidimensional or not. A fundamental assumption of Cronbach’s alpha is that the items in a scale measure a single, unidimensional construct.

The Anthropocene Awareness Scale was optimized through correlation, reliability, and factor analysis. To ensure a reliable set of highly intercorrelated items, each item should show a strong correlation with the rest of the scale items. The corrected item-total correlation assesses this by correlating the target item with the scale’s total score, excluding the item itself. Evaluating corrected item-total correlations is recommended, as higher values indicate that the item is better aligned with the overall construct being measured. Although the threshold of 0.30 is not a strict criterion, it is widely used in psychometric practice, especially during the exploratory phase of scale development [17, 22]. In Model 1, which initially included 15 items, five items were removed due to corrected item-total correlations below 0.30. In Model 2, reduced to 10 items, one additional item was excluded based on the same criterion. While reviewing the nine items in Model 3, one item (Item 8 from the initial Anthropocene Awareness scale; see S1 Table) was found to be double-barreled and was therefore removed. Therefore, double-barreled items can be problematic as they may be interpreted ambiguously, making it unclear which concept respondents are endorsing [17]. The item conflated two distinct concepts: predictability (the ability to foresee outcomes) and controllability (the capacity to influence outcomes). Even from a statistical perspective, the item exhibited relatively lower item-total correlation and factor loading, indicating a poor fit with the overall scale. As a result, the scale was optimized, yielding a final version with eight items and a Cronbach’s alpha of 0.887. Details on the changes in corrected item-total correlations and coefficient alpha during the scale optimization process are provided in S2 Table. At the same time, the factor loadings from the principal component analysis are presented in S3 Table.

### 3.2. Measures

#### 3.2.1. Anthropocene Awareness Scale

Table 1 presents the eight items used to assess awareness of the Anthropocene. They are worded so that respondents who agree with the statement also express awareness of the Anthropocene. Respondents were assigned scores on a 5-point Likert scale, with 5 representing “Strongly Agree,” 4 representing “Mildly Agree,” 3 representing “Unsure,” 2 representing “Mildly Disagree,” and 1 representing “Strongly Disagree.”

**Table 1:** Frequency distributions and corrected item-total correlations for Anthropocene Awareness Scale items.

Do you agree or disagree that:	SA^b^	MA	U	MD	SD	Mean	S.D.	r_i-t_
1. Humans have a dominant impact on the Earth beyond cities and regions.	24.8%	45.4%	24.4%	4.8%	0.8%	3.89	0.859	0.677
2. The rapid increase in human socioeconomic activities, such as climate change, has caused the Anthropocene crisis.	16.6	50.1	28.3	4.5	0.6	3.78	0.794	0.649
3. The human impact on the Earth is accelerating at a rapid pace.	25.4	47.8	21.9	4.1	0.7	3.93	0.836	0.716
4. The human impact on the Earth exceeds the limit of what the planet can afford.	19.4	47.6	26.8	5.5	0.8	3.79	0.843	0.706
5. Catastrophes that take place on Earth are severe to an irreversible extent.	18.7	48.6	27.0	5.0	0.6	3.80	0.820	0.681
6. If the current situation continues, numerous species, including humans, will go extinct.	23.4	45.1	26.4	4.3	0.9	3.86	0.853	0.695
7. Humankind is not the ruler of nature but merely a species with its capabilities and limitations.	14.0	45.3	34.0	5.6	1.1	3.66	0.828	0.477
8. Humans should blend in with nature and machines rather than control them.	27.1	43.3	24.7	4.4	0.6	3.92	0.859	0.669

a Question wording: “Listed below are statements about the relationship between humans and the Earth. For each one, please indicate whether you STRONGLY AGREE, MILDLY AGREE, are UNSURE, MILDLY DISAGREE, or STRONGLY DISAGREE with it.”

b SA = Strongly Agree, MA = Mildly Agree, U = Unsure, MD = Mildly Disagree, and SD = Strongly Disagree, S.D. = Standard Deviation, r_i-t_ = Corrected Item-Total Correlations

### 3.2.2. Other factors

Psychological and socioeconomic factors identified in previous studies on environmental attitudes and behaviors were included in the survey. First, environmental attitude is assessed using a 5-point Likert scale (1 = Strongly disagree, 5 = Strongly agree). The sum of fifteen items was used to measure participants’ environmental attitudes. The reported Cronbach’s alpha of the scale was 0.83, and Cronbach’s alpha for this study was 0.88. Second, we asked about five pro-environmental behavior variables including recycling, waste reduction, energy conservation, and water conservation, a self-report of pro-environmental behavior variable assessed using a 5-point Likert scale. Third, we hypothesized that individuals who have heard of the Anthropocene would show higher scores on the Anthropocene Awareness Scale compared to those who have not. To test this hypothesis and examine the validity of the proposed Anthropocene Awareness Scale, we included the question: “Have you ever heard of the Anthropocene?” This variable is named “knowledge” (0 = No, 1 = Yes).

We included four socioeconomic factors: age, gender, income, and level of education. Age, income, and education level are treated as continuous variables. The average monthly wage is used to measure income, and it is coded as “1 = Below 1 million KRW” and “11 = Over 10 million KRW.” Gender is represented as “1 = Men” and “2 = Women.”

### 3.3. Analyses

Descriptive statistics, such as frequency distribution and corrected item-total correlations, were calculated for the Anthropocene Awareness Scale to examine the data. Second, reliability analysis is conducted to evaluate the reliability of the Anthropocene Awareness Scale. Third, factor analysis is conducted to evaluate the dimensionality of the Anthropocene Awareness Scale. In this study, principal component analysis is conducted for factor analysis. Fourth, correlation analysis examines the correlation between the Anthropocene Awareness Scale and other factors, such as the New Ecological Paradigm (NEP) scale, environmental behavior, and socioeconomic factors. All analyses were conducted using SPSS v.29 statistical software, and all statistical tests were evaluated at p < .05, two-tailed.

## 4. Results

### 4.1. Descriptive statistics

Table 1 gives each item’s frequency distribution, mean, standard deviation, and corrected item-total correlation. The frequency distributions for the items reveal that the Anthropocene awareness is widely accepted among the public. The majority of participants agreed with the Anthropocene Awareness Scale items, with agreement levels (“strongly” and “mildly” agree) ranging from 59.3% for item 7 to 73.2% for item 3. Other items that received strong perception are item 1 and item 8, as over 70 percent “strongly” and “mildly” agreed with both.

A mean close to the center of the range of possible scores is desirable for the scale. In other words, the mean of each item near 3 would be ideal because the response options for each item ranged from 1 to 5. If the average score on an item is close to one of the extreme ends of the possible range of scores, then the item may not be able to include specific construct values. The frequency pattern of pro-Anthropocene Awareness responses is reflected in the item means. The item means of the scale is 3.83, and the means range from a low of 3.66 for item 7 to a high of 3.93 for item 3. It implies that the scale items are appropriate in terms of the mean.

The responses to the Anthropocene awareness items suggest a high level of awareness of the Anthropocene among Koreans. In other words, the data on response distributions to the Anthropocene awareness items show that the Korean public is more accepting of the facets of the Anthropocene than we had expected. Concepts such as the Earth system, the Great Acceleration, planetary boundaries, the sixth extinction, and multispecies relations are gradually becoming more ingrained in the perception of the Korean public.

### 4.2. Reliability and dimensionality of the Anthropocene Awareness Scale

We assumed that all eight items in Table 1 measure various aspects of Anthropocene awareness and investigated whether combining these items into a single scale is appropriate. The last column of Table 1 shows the corrected item-total correlations. These correlations are positive and large in magnitude, meaning that each item is strongly related to the overall scale score. The corrected item-total correlations for the sample range from .477 to .716, and the average of .658. The high correlations between items in the scale suggest that the scale is reliable and has high internal consistency. Cronbach’s alpha, a measure of internal consistency, is .887 for the scale, which means that the items on the scale are highly correlated with each other. This suggests that the scale is measuring a single underlying construct. The dimensionality of the scale is suggested by the results of the principal factor analysis. There is only one principal factor, and it accounts for 51.9 percent of the variance in the sample (Table 2). Further, all nine items load highly on the factor, ranging from .458 to .794 and averaging .712. The results show that all nine items of the Anthropocene Awareness Scale are internally consistent and unidimensional.

**Table 2:** Principal components analysis of Anthropocene Awareness Scale items with varimax rotation.

	Factor 1
AA1	0.765
AA2	0.738
AA3	0.799
AA4	0.791
AA5	0.769
AA6	0.782
AA7	0.572
AA8	0.758
Eigenvalue	4.498
Percentage of variance (%)	56.22

Note. AA = Anthropocene Awareness.

## 5. Discussion

### 5.1. Validity of the Anthropocene Awareness Scale

Having combined the Anthropocene Awareness Scale items into a single scale, we must assess its validity. This means verifying whether the scale accurately measures the intended construct. We examined the scale’s criterion-related, content, and construct validity for this purpose. First, to assess the concurrent validity of the Anthropocene Awareness Scale, a type of criterion-related validity, we can look at several pieces of information. The Anthropocene awareness items seem to tap into people’s primitive beliefs about the relationship between humans and the planet. Rokeach [40] defined primitive beliefs as the core of a person’s belief system or their basic truths about the world and themselves. These beliefs are thought to influence a wide range of other beliefs and attitudes, including those about environmental issues [22, 41]. In short, the Anthropocene awareness, reflected by a high score on the Anthropocene Awareness Scale, should lead to environmental attitudes and behaviors.

In this context, the relationship between scores on the Anthropocene awareness and other measures of environmental attitudes implied the concurrent validity of the Anthropocene Awareness Scale. First, the questionnaire included the NEP scale, a representative measurement instrument for environmental attitudes, and respondents were asked to indicate how they agreed with the NEP. The responses to NEP were strongly correlated (r = .65, p < .01). Participants reported their frequency of engaging in environmental behaviors. The four environmental behaviors were found to be internally consistent (alpha = .760), and their responses to the 8-item Anthropocene Awareness Scale were also positively correlated (r = .41, p < .01). To assess the concurrent validity of the Anthropocene Awareness Scale, we employed two measures. We hypothesized that individuals who score higher on the Anthropocene Awareness Scale would exhibit greater eco-friendliness and engagement in environmental behaviors.

It is harder to assess the content validity because it depends on experts agreeing that the items on the scale adequately cover the concept being measured [17]. We tried to include a variety of items that measure all the important aspects of the Anthropocene. We carefully considered the literature on the Anthropocene and also consulted with experts to choose the items for the scale. Additionally, we found some evidence related to construct validity. Previous research has shown that education and income are two of the most important factors influencing people’s environmental attitudes. S4 Table shows that higher income and education levels correlate with higher Anthropocene awareness scores, which we would expect. They are more likely to learn about the ideas underlying the Anthropocene in college, from the news, and from reading books and articles, and to understand complicated facets of the Anthropocene. The correlation coefficients imply that the Anthropocene Awareness Scale has construct validity.

### 5.2. Implications and potential applications

We propose a new scale grounded in the Anthropocene studies. Words have power only when they are widely accepted [16]. The development of the Anthropocene Awareness Scale can intensify the analytical potential and the plasticity of the Anthropocene concept. The predicaments of the Anthropocene require a critical consideration of resources and ideas from various fields, and scientists, activists, and citizens should be gathered on a large scale. The political influence of the Anthropocene comes from the analytical potential to bring together researchers across the natural sciences, social sciences, humanities, and arts sectors.

The plasticity of the concept of the Anthropocene can encompass various fields and public interests. This term will promote interdisciplinary cooperation by enabling researchers from diverse backgrounds to explore further political, cultural, and ecological dimensions of the Anthropocene’s challenges. Individual attitudes and behaviors are still important in the Anthropocene, but little research has explored the extent to which the public has adopted Anthropocene perspectives, empirically. We believe that the Anthropocene Awareness Scale has the potential to appeal to quantitative researchers. For example, sociologists, psychologists, and political scientists may use the scale in social-political-psychological models of individual attitudes and behaviors from the perspective of the Anthropocene. Furthermore, the scale is likely to facilitate empirical studies based on the Anthropocene, and such pieces of evidence may impact environmental policies. Policy researchers can also directly conduct a survey, including the scale. Government-funded research institutes may consider containing the scale in a national survey. The results of these surveys could support formulating the new environmental agenda in the Anthropocene. As a post-test, the scale could provide information on the effect of specific lectures, education, public information campaigns, and exhibitions related to the Anthropocene.

Recent studies in environmental education related to the concept of the Anthropocene provide valuable insights for exploring potential applications of the Anthropocene Awareness Scale in educational contexts [42–44]. By creating a comprehensive competence framework aligned with the European Green Deal, the Green SCENT project emphasizes the integration of key sustainability competencies—encompassing knowledge, skills, and attitudes—across various educational settings [42, 44]. Tomassi et al. [44] position their Competence Framework within the Anthropocene discourse, emphasizing the urgent need for decision-making based on sustainability, equity, and justice while addressing the complex interlay between the Anthropocene and climate change. Lee and Park [43] explore how the Anthropocene concept can be effectively integrated into science education to enhance students’ critical understanding of human impacts on Earth. They introduce the concept of Anthropocene literacy, which involves grasping the nature of science through the Anthropocene lens, adopting a multidisciplinary approach, and examining the socio-environmental implications of human activities. The proposed educational framework aims to equip students with the knowledge and critical thinking skills needed to navigate the complexities of this epoch. The Anthropocene Awareness Scale, tailored to different educational levels, has the potential to serve as a flexible tool for educators to foster critical reflections on human impacts. However, translating these interdisciplinary insights into a practical educational resource is complex and requires careful adaptation to prevent oversimplification. Instead of being a definitive assessment tool, the scale might be better positioned as a catalyst for interdisciplinary learning, helping educators guide people through the nuanced challenges of the Anthropocene.

### 5.3. Limitations and future research directions

We developed and administered a scale to measure Anthropocene awareness among the Korean public. This study provides valuable empirical insights into individuals’ awareness of the Anthropocene; however, these findings should be interpreted considering the scale’s limitations. While the Anthropocene Awareness Scale shows promise in gauging public perceptions, its validity requires further research. Although internal consistency reliability is well-established, additional studies and expert reviews are needed to strengthen its validity in the future. Future research could consider conducting Confirmatory Factor Analysis (CFA) to assess the structural validity of the scale further. Using CFA would offer a more detailed evaluation of its factor structure, strengthening the measurement tool and providing more precise insights for studies looking to apply.

The results indicate a possible association between the Anthropocene Awareness Scale and sociodemographic factors such as income and education, suggesting a broad spectrum of human understanding regarding the Anthropocene. Future studies could aim to validate the Anthropocene Awareness Scale across different cultural contexts. Although this study was conducted in South Korea, interpretations of the Anthropocene may differ depending on each region’s historical and socioeconomic conditions. To evaluate the scale’s applicability and gain additional insights, it would be valuable to test it across diverse cultural settings.

A further focus for future research could be to investigate the scale’s relationship with other environmental attitudes and behaviors, aiming to enhance its practical relevance. Our findings reveal a correlation between the Anthropocene Awareness Scale, NEP, and general environmental behaviors; however, further research is necessary to explore the causal relationship between the Anthropocene Awareness Scale and specific environmental behaviors. To this end, exploring the practical implications of this scale for environmental interventions is crucial.

## 6. Conclusions

The political influence of the Anthropocene concept stems from its analytic potential to encompass various disciplines and public attention. The discussion of the Anthropocene, which began among Earth system scientists and geologists, is rapidly spreading to various fields. However, there is a lack of research examining the extent to which the public has adopted the views embodied in the Anthropocene. We developed a new scale to measure awareness of the Anthropocene, drawing on recent Anthropocene discourse. The political influence of the Anthropocene lies in its analytical potential to bring together researchers across the disciplines. We believe quantitative researchers will find this scale useful in examining social-political-psychological models of individual attitudes and behaviors. Furthermore, the Anthropocene Awareness Scale facilitates empirical studies on the Anthropocene using survey data, potentially informing environmental policy decisions. We hope that the Anthropocene Awareness Scale will be used and discussed in future studies, which will help to improve the scale and encourage more discussion about individual attitudes and behaviors in the Anthropocene.

## Supporting information

S1 Table The initial item pool for the Anthropocene Awareness Scale. (DOCX)

S2 Table Corrected item-total correlation from reliability test. (DOCX)

S3 Table Factor loadings and eigenvalues from factor analysis. (DOCX)

S4 Table Pearson correlation matrix for the Anthropocene Awareness Scale, psychological, and sociodemographic variables. (DOCX)
