# Supplementary material for: The development of Anthropocene Awareness Scale
Source: PLoS One. 2025 Feb 6;20(2):e0316315. doi: 10.1371/journal.pone.0316315 (PMC11801584; doi:10.1371/journal.pone.0316315)
Supplement: S1 Table — (DOCX) [file pone.0316315.s001.docx]

| **S1 Table.**  *The Initial Item Pool for The Anthropocene Awareness Scale* | |
| --- | --- |
| No. | Items. |
| 1. | Humans have power comparable to that of nature. |
| 2. | Humans have a dominant impact on the Earth beyond cities and regions. |
| 3. | Humans use nature and pursue economic growth. |
| 4. | The rapid increase in humans' socioeconomic activities has caused the Anthropocene crisis, such as climate change. |
| 5. | The human impact on the Earth is accelerating at a rapid pace. |
| 6. | Humans can rule or control nature completely. |
| 7. | The human impact on the Earth exceeds the limit of what the planet can afford. |
| 8. | The crisis of the Anthropocene is unpredictable and uncontrollable. |
| 9. | Human abilities can recover the Earth damaged by humans. |
| 10. | Catastrophes that take place on Earth are severe to an irreversible extent. |
| 11. | If the current situation continues, numerous species, including humans, will go extinct. |
| 12. | Humans have the capabilities to stabilize climate and protect the Earth. |
| 13. | Humankind is not the ruler of nature but merely a species with its capabilities and limitations. |
| 14, | Humans should blend in with nature and machines rather than control them. |
| 15. | Humans can predict and solve the crisis of the Anthropocene by using science and technology. |
