# Supplementary material for: The development of Anthropocene Awareness Scale
Source: PLoS One. 2025 Feb 6;20(2):e0316315. doi: 10.1371/journal.pone.0316315 (PMC11801584; doi:10.1371/journal.pone.0316315)
Supplement: S2 Table — (DOCX) [file pone.0316315.s002.docx]

| **S2 Table.** | | |  |  |
| --- | --- | --- | --- | --- |
| *Corrected Item-Total Correlation from Reliability Test* | | |  |  |
|  | Model 1 (n=15) | Model 2 (n=10) | Model 3 (n=9) | Model 4 (n=8) |
| AA1 | -0.472 | - | - | - |
| AA2 | 0.538 | 0.664 | 0.664 | 0.677 |
| AA3 | 0.002 | - | - | - |
| AA4 | 0.519 | 0.638 | 0.651 | 0.649 |
| AA5 | 0.59 | 0.715 | 0.709 | 0.716 |
| AA6 | 0.313 | 0.229 | - | - |
| AA7 | 0.593 | 0.698 | 0.703 | 0.706 |
| AA8 | 0.311 | 0.338 | 0.376 | - |
| AA9 | 0.127 | - | - | - |
| AA10 | 0.586 | 0.672 | 0.688 | 0.681 |
| AA11 | 0.578 | 0.681 | 0.688 | 0.695 |
| AA12 | -0.071 | - | - | - |
| AA13 | 0.402 | 0.467 | 0.489 | 0.477 |
| AA14 | 0.541 | 0.667 | 0.654 | 0.669 |
| AA15 | 0.022 | - | - | - |
| Cronbach’s alpha | 0.652 | 0.855 | 0.879 | 0.887 |
