# Supplementary material for: The development of Anthropocene Awareness Scale
Source: PLoS One. 2025 Feb 6;20(2):e0316315. doi: 10.1371/journal.pone.0316315 (PMC11801584; doi:10.1371/journal.pone.0316315)
Supplement: S3 Table — (DOCX) [file pone.0316315.s003.docx]

| **S3 Table.** | | |  |  |  |  |  |
| --- | --- | --- | --- | --- | --- | --- | --- |
| Factor Loadings and Eigenvalues from Factor Analysis | | | | | | | |
|  | Model 1  (n=15) | | | Model 2  (n=10) | | Model 3 (n=9) | Model 4 (n=8) |
|  | 1 | 2 | 3 | 1 | 2 | 1 | 1 |
| AA1 | -0.321 | -0.498 | 0.516 | - | - | - | - |
| AA2 | 0.755 | -0.133 | -0.128 | 0.761 | 0.114 | 0.756 | 0.765 |
| AA3 | -0.076 | 0.42 | -0.382 | - | - | - | - |
| AA4 | 0.724 | -0.069 | 0.079 | 0.732 | -0.079 | 0.738 | 0.738 |
| AA5 | 0.801 | -0.06 | -0.09 | 0.8 | 0.11 | 0.794 | 0.799 |
| AA6 | 0.378 | 0.456 | -0.523 | 0.326 | 0.782 | - | - |
| AA7 | 0.782 | -0.028 | 0.028 | 0.789 | 0.027 | 0.789 | 0.791 |
| AA8 | 0.411 | 0.224 | 0.71 | 0.427 | -0.597 | 0.458 | - |
| AA9 | 0.003 | 0.794 | 0.043 | - | - | - | - |
| AA10 | 0.756 | 0.036 | 0.163 | 0.766 | -0.075 | 0.772 | 0.769 |
| AA11 | 0.771 | -0.029 | 0.008 | 0.777 | 0.025 | 0.777 | 0.782 |
| AA12 | -0.227 | 0.744 | 0.128 | - | - | - | - |
| AA13 | 0.555 | 0.054 | 0.253 | 0.562 | -0.275 | 0.577 | 0.572 |
| AA14 | 0.759 | -0.082 | -0.157 | 0.759 | 0.183 | 0.748 | 0.758 |
| AA15 | -0.122 | 0.711 | 0.019 | - | - | - | - |
| Eigenvalues | 4.927 | 2.756 | 1.094 | 4.744 | 1.112 | 4.670 | 4.498 |
| *Note.* Extraction Method: Principal Component Analysis. Rotation Method: Oblimin with Kaiser Normalization. | | | | | | | |
