# Supplementary material for: The development of Anthropocene Awareness Scale
Source: PLoS One. 2025 Feb 6;20(2):e0316315. doi: 10.1371/journal.pone.0316315 (PMC11801584; doi:10.1371/journal.pone.0316315)
Supplement: S4 Table — (DOCX) [file pone.0316315.s004.docx]

**S4 Table.**

*Pearson Correlation Matrix for the Anthropocene Awareness Scale, Psychological, and Sociodemographic Variables*

|  | AA | NEP | Behavior | Age | Education | Income |
| --- | --- | --- | --- | --- | --- | --- |
| AA | 1 |  |  |  |  |  |
| NEP | .645^**^ | 1 |  |  |  |  |
| Behavior | .408^**^ | .213^**^ | 1 |  |  |  |
| Age | .028 | .139^**^ | .153^**^ | 1 |  |  |
| Education | .085^**^ | .014 | .029 | -.435^**^ | 1 |  |
| Income | .119^**^ | .049^*^ | .088^**^ | -.136^**^ | .325^**^ | 1 |

*Note. AA=Anthropocene Awareness Scale. NEP=New Ecological Paradigm Scale.*

** Correlation is significant at the 0.05 level (2-tailed).*

*** Correlation is significant at the 0.01 level (2-tailed).*
